# Penetrating Keratoplasty versus Deep Anterior Lamellar Keratoplasty for Keratoconus: A Systematic Review and Meta-analysis

**DOI:** 10.18502/jovr.v17i1.10174

**Published:** 2022-01-21

**Authors:** Majid Shams, Ali Sharifi, Zahra Akbari, Ali Maghsoudlou, Mohammad Reza Tajali

**Affiliations:** ^1^Department of Ophthalmology, Shafa Hospital, Kerman University of Medical Sciences, Kerman, Iran; ^2^Student Research Committee, Kerman University of Medical Sciences, Kerman, Iran; ^3^Noor Eye Hospital, Tehran, Iran

**Keywords:** Corneal Transplant, Deep Anterior Lamellar Keratoplasty, Keratoconus, Penetrating Keratoplasty

## Abstract

Keratoconus is the most common form of primary corneal thinning. Different methods have been suggested to deal with the condition, including glasses, contact lenses, and surgical interventions, like penetrating keratoplasty (PKP) and deep anterior lamellar keratoplasty (DALK), well-known methods of the latter. This study was conducted to compare the outcomes and side effects of the two mentioned keratoplasty techniques. First, we systematically reviewed all original articles studies on PubMed, Scopus, Web of Science, and Embase. Then, the extracted data were pooled and meta-analyzed on each of the intended outcomes. A total of 30 studies were included in which PKP was more commonly performed compared to DALK. We found that adverse outcomes consisting of cataracts, graft rejection, graft failure, High-IOP, and corneal infection, were all more common findings in the PKP groups compared to the DALK groups. However, only for the high-IOP, cataracts, and graft rejection, the analysis of the extracted results demonstrated statistical significance. Overall, the DALK groups demonstrated significantly better results when considering the improvement levels by measuring the Endothelial Cell Count (ECC) and Spherical Equivalent (SE). In addition, though statistically insignificant, the Central Corneal Thickness(CCT), Best Corrected Visual Acuity(BCVA), Topographic Cylinder(TC), Refractive Cylinder values were greater in the PKP groups. Based on our study and with its limitations in mind, we can conclude that DALK can be a relatively safer and more effective procedure. Though, a larger number of high-standard randomized clinical trials still need to be conveyed for more definite conclusions.

##  INTRODUCTION

Keratoconus defined as bilateral^[1,2]^ and asymmetrical^[3,4]^ degeneration of the cornea is the most common form of primary corneal thinning. Local thinning of the cornea caused by keratoconus leads to corneal protrusion and then severe myopia and irregular astigmatism.^[2,5]^

Different methods have been utilized to treat this condition, including prescribing glasses and contact lenses for the early stages^[5,6]^ and keratoplasty for the advanced stages of the disease.^[6]^ Keratoconus is the most common pathology requiring corneal transplants in most ophthalmology centers worldwide. Similarly, based on available data, keratoconus is also the most common eye pathology requiring corneal transplant in Iran.^[8,9]^ According to one study, approximately 10–20% of keratoconus cases end up requiring standard penetrating keratoplasty (PKP). If the corneal cone's size, the severity of the keratoconus or corneal hydrops limits the possibility of utilizing contact lenses to treat the condition, keratoplasty should be performed.^[10]^ Despite the popular use and high success rates of PKP, there is always a 20% risk that the host develops an immune reaction to the graft, of which 85% is due to the endothelial cell rejection. Approximately 2.5% of graft rejections lead to graft failure.^[11,12]^

Some studies have also shown that in PKP cases, the number of endothelial cells decreases by 4.2% each year. This decline may continue until 5–10 years after the transplantation.^[13]^ Other PKP complications include expulsive hemorrhage, endophthalmitis, synechiae of the iris to the angle or point of incision of the graft, side effects of long-term corticosteroids use, and predisposition to traumatic injuries.^[14]^

In the recent decade, the rate of performing the lamellar keratoplasty (LK) procedure has increased.^[15]^ Potential immunological incompatibilities after the insult, leading to complications including graft rejection, are of considerable importance, therefore, the injured corneal layers are removed, and the healthy tissue is preserved.^[16]^ Deep anterior lamellar keratoplasty (DALK) is a type of LK that reconnects the stroma to the Descemet's membrane in cases whose stroma might be in danger of loss.^[17]^ This technique prevents the recipient's endothelium replacement with the donor's and mitigates the risk of endothelial induced rejection. However, the risk of rejection will not be completely eliminated due to the remaining epithelial layer.^[18,19]^

Although the LK is rapidly becoming the method of choice in corneal transplant, some studies have compared DALK with PKP to determine the more appropriate option for treating keratoconus.^[20]^ Considering the importance of the subject and that very few comprehensive studies have evaluated and compared both techniques in keratoconus cases, our study aims to conduct a systematic review to compare the outcomes and side effects of these two techniques.

##  METHODS

We completed our systematic review in accordance with the Preferred Reporting Items for Systematic Reviews and Meta-Analyses (PRISMA) guidelines (http://www.prisma-statement.org/).^[21]^

For this systematic review, we searched PubMed, Scopus, Embase, Web of Science databases for articles published up to the end of May 2021.

### Inclusion and Exclusion Criteria

All English comparative studies on adults including clinical trials, retrospective and prospective cohort studies on keratoconus treated with DALK and PKP were used in the data assessment. We excluded all editorials, conferences, commentaries, letter to editors, and reviews. In addition, non-English studies, case reports, case–controls, noncomparative studies, and those evaluating DALK and PKP effects without a focus on keratoconus were excluded. There were no limitations regarding the sex of the evaluated cases. However, only studies which evaluated adults were included.

### Search Strategy

We conducted a thorough manual search on the Web of Science, Embase, PubMed, and Scopus, considering the publications up to May 2021. The searched queries are delineated below:

### SCOPUS and Web of Science

TITLE-ABS-KEY (compar* AND (lamell* AND penet*) AND keratocon*) AND (LIMIT-TO (DOCTYPE, "ar")) AND (LIMIT-TO (LANGUAGE, "English"))

### Pubmed

compar*[tiab] AND (lamell*[tiab] AND penet*[tiab]) AND keratocon*[tiab] Filters: Clinical Study, Clinical Trial, Comparative Study, Controlled Clinical Trial, Evaluation Study, Multicenter Study, Observational Study, Pragmatic Clinical Trial, Randomized Controlled Trial, English

### Embase

compar*:ti,ab,kw AND lamell*:ti,ab,kw AND penet*:ti,ab,kw AND keratocon*:ti,ab,kw AND ('case study'/de OR 'clinical trial'/de OR 'cohort analysis'/de OR 'comparative effectiveness'/de OR 'comparative study'/de OR 'controlled clinical trial'/de OR 'controlled study'/de OR 'cross sectional study'/de OR 'intervention study'/de OR 'major clinical study'/de OR 'observational study'/de OR 'prospective study'/de OR 'randomized controlled trial'/de OR 'randomized controlled trial topic'/de OR 'retrospective study'/de) AND 'article'/it

### Evaluating Recovered Evidence

After completing the search, two reviewers separately removed duplicated findings via Endnote version 20. A manual check for duplication was also performed to ensure none existed. Subsequently, two authors performed initial evaluations of titles and abstracts of the recovered evidence. After recovering all the available articles in the second phase, they were then evaluated by the research team.

### Data Extraction

Two authors independently extracted data from the articles according to the following criteria:

∙ Included study's first author

∙ The year the study was published

∙ Study type

∙ Country of origin

∙ Patients' age and sex

∙ Duration of follow-up

∙ Corneal infection rates

∙ Graft rejection rates (the rate of the rejection episodes seen in cases)

∙ Graft failure rates

∙ Cataract rates

∙ High-intraocular pressure (IOP; also known as ocular hypertension; an eye pressure of > 21 mm Hg) rates

∙ Mean and standard deviation (SD) of best-corrected visual acuity (BCVA) in LogMAR scale (defined as the highest score on the Snellen chart when wearing either a visual aiding device, like glasses or contact lenses)

∙ Refractive (RC) and topographic cylinder (TC; defined as refractive power of the cylindrical lenses and the mapping the anterior curvature of the cornea, respectively)

∙ Central corneal thickness (CCT; defined as the thickness of the cornea measured by optical low coherence reflectometry)

∙ Endothelial cell count (ECC; estimation of corneal endothelial reserve by corneal endothelial photography)

∙ Spherical equivalent (an estimate of the eyes' refractive error calculated by merging the nearsightedness or farsightedness and cylindrical astigmatism components)

All the mentioned values were included based on the last known follow-up of each study. Finally, the extracted data were reviewed and double-checked by the senior author.

**Table 1:** Data related to evaluated studies


**Author**	**Design**	**Year**	**Country**	**Sample size PKP**	**Sample size DALK**	**Mean age in PKP**	**Mean age in DALK**	**Follow-up PKP (month)**	**Follow-up DALK (month)**	**PKP Sex Male**	**PKP Sex Female**	**DALK Sex Male**	**DALK Sex Female**	**Main value evaluated**
Abdelaal et al^[23]^	Retrospective cohort	2021	Saudi Arabia	15	21					Graft Rejection
Abu Eta et al^[24]^	Retrospective cohort	2020	Israel	21	32					High-IOP
Akdemir et al^[25]^	Retrospective cohort	2012	Turkey	30	30	28.07	29.67	12.58	12.58	16	14	14	16	BCVA
Alzahrani et al^[26]^	Retrospective cohort	2018	UK	21	16	30.14	32.56	13.95	14.87	14	7	11	5	BCVA, CCT
Amayem et al^[27]^	Retrospective cohort	2012	Saudi Arabia	30	47	26.5	24.3	24	24			Refractive Cylinder, SE
Bahar et al^[28]^	Retrospective cohort	2008	Canada	20	17	42.2	32.5	53.2	17	11	9	11	6	Endothelial Cell Count, Infection Rate, Graft Rejection, Topographic Cylinder
Cohen et al^[29]^	Retrospective cohort	2010	USA	30	11	35.4	45.5	21.9	22.5	21	9	8	3	Cataract Frequency, High-IOP, Graft Rejection, Refractive Cylinder, SE, BCVA
Donoso et al^[30]^	Retrospective cohort	2015	Chile	49	90	31.7	28.3	48.6	36.8			High-IOP, ECC, Infection Rate, Graft Rejection, Topographic and Refractive Cylinder, CCT
Funnell et al^[31]^	Cohort	2006	UK	20	20	32	28	12	12	14	6	11	9	High-IOP, Graft Rejection, Graft Failure, Topographic Cylinder
Godefrooij et al^[32]^	Retrospective cohort	2016	Netherlands	736	297	38.07	35.65		499	237	204	93	BCVA
Hamdi et al^[33]^	Cross-sectional	2017	Egypt	12	24	26.95	23.79		9	3	14	10	Topographic and Refractive Cylinder, SE, BCVA
Huang et al^[34]^	Retrospective cohort	2015	China	79	68	24.3	24.1	93.1	93.5	50	29	40	28	Topographic and Refractive Cylinder, SE, BCVA
Jafarinasab et al^[35]^	Cross-sectional	2011	Iran	45	23	29.8	27.2	31.4	29.2			Topographic Cylinder, SE, BCVA
Janiszewska-Bil et al^[36]^	Cohort	2021	Poland	40	50	28.4	28.6	12	12	24	16	28	22	High-IOP, ECC, Topographic Cylinder, CCT, BCVA
Javadi et al^[37]^	Randomized clinical trial	2010	Iran	35	42	30.89	26.91	24.6	22	28	7	29	13	Graft Rejection, Topographic Cylinder, CCT, SE, BCVA
Jones et al^[38]^	Retrospective cohort	2009	UK	1917	455					Graft Rejection, Graft Failure, SE, BCVA
Kasbekar et al^[39]^	Cohort	2014	UK	3124	1086		60	60			Infection rate, Graft Rejection, Graft Failure
Khattak et al^[40]^	Retrospective cohort	2018	Saudi Arabia	99	108	28.9	27.8	29.3	28.1	59	40	67	41	High-IOP, ECC, Cataracts, Infection rate, Graft Rejection, Graft Failure, Topographic and Refractive Cylinder, SE, BCVA
Kim et al^[41]^	Retrospective cohort	2011	Korea	38	19	26.2	25.3	51.7	22.6	25	13	17	2	SE
Koytak et al ^[42]^	Retrospective cohort	2011	Turkey	39	44	36.24	34.54	24	24	24	15	26	18	ECC, CCT
Kubaloglu et al ^[43]^	Cohort	2011	Turkey	24	20	30.2	25.6	6	6		112	95	Topographic and Refractive Cylinder, SE, CCT, BCVA
Macintyre et al ^[44]^	Retrospective cohort	2014	Australia	42	31	32.3	29.2	53.7	51.8	22	20	19	12	Cataracts, Graft Rejection, Graft Failure, Refractive Cylinder, BCVA
Motlagh et al ^[45]^	Cross-sectional	2012	Iran	57	49		35	30.3			SE, BCVA
Oh et al ^[46]^	Retrospective cohort	2013	Korea	5	11	27.4	28	45	30	3	2	7	4	ECC, Refractive and Topographic Cylinder, CCT, SE, BCVA
Pedrotti et al ^[47]^	Retrospective cohort	2016	Italy	16	16	34.1	35.9		7	9	7	9	Graft Rejection, Graft Failure, Topographic and REfractive Cylinder, CCT, SE, BCVA
Sogutlu Sari et al ^[48]^	Cohort	2012	Turkey	75	99	28.44	27.59	25.53	21.54			Graft Rejection, Refractive Cylinder, SE, BCVA
Watson et al ^[49]^	Retrospective cohort	2004	UK	22	25	33.9	32.6	55	28	14	8	17	8	Infection, Graft Rejection, Graft Failure,
Yüksel et al ^[50]^	Clinical trial	2017	Turkey	38	38	35.3	34.9	12	12	15	23	10	28	High-IOP, Graft Rejection, Topographic Cylinder, SE, BCVA
Zhang et al ^[51]^	Retrospective cohort	2013	China	52	75	21.9	20.6	60.2	46.9	45	7	55	20	High-IOP, Cataracts, Graft Rejection, Topographic Cylinder, SE
Ziaei et al ^[52]^	Retrospective cohort	2019	New Zealand	42	27	35	36.1		25	17	12	15	CCT

**Figure 1:**
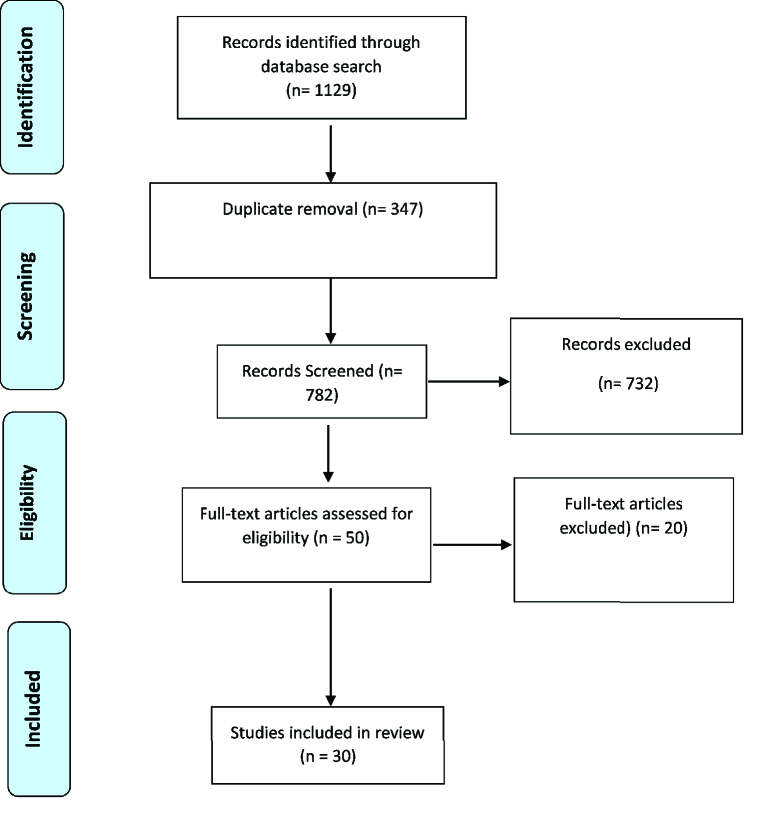
Diagram of the studies evaluation.

### Quality Assessment

The quality of the included studies was evaluated independently by two authors using the critical appraisal tool, provided by The Joanna Briggs Institute, containing 13, 12, and 8 items for assessing RCTs, cohort, and cross-sectional studies, respectively.

### Data Analysis

For quantitative outcomes, the mean differences and SD, and the risk-ratio for qualitative outcomes were determined and analyzed. Methods of meta-analysis and random-effects models were used to combine the results using the 14 ${}^{\mathrm{th}}$ edition of the STATA software. Furthermore, heterogeneity between the studies was determined by employing the I^2^ test. The *p*-value was set at < 0.05 for the significance level.

##  RESULTS

We identified 1129 articles in the systematic search of resources. After reviewing the titles and abstracts, 1079 articles were excluded from the study of which 347 were duplications. After reviewing the full-text of the articles, 20 were put aside again. Finally, 30 articles were included into this meta-analysis. The information of the selected articles is shown in Figure 1.

Among the 30 articles chosen, 25 were cohort (retrospective or prospective), 3 were cross-sectionals, and 2 were randomized-clinical trials. Information about each study is shown in Table 1.

### Quality Assessment

Based on the Joanna Briggs Institute's critical appraisal tool, the three cross-sectional studies scored within a range of 6–8 out of a possible 8. The two randomized-clinical trials scored 10 out of a possible 13. In addition, the 25 cohort studies within a range of 9–11 out of a possible 12.

### Meta-Analysis Results

#### Central corneal thickness (CCT)

**Figure 2:**
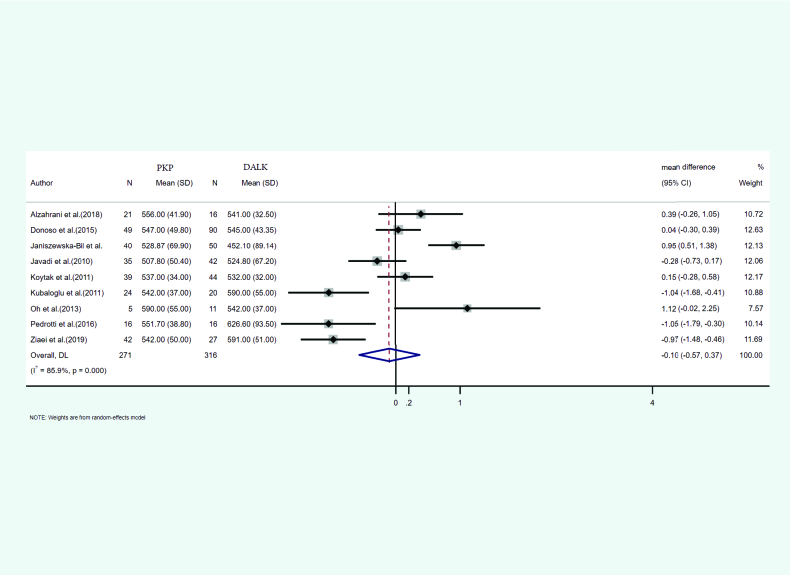
Results of meta-analysis for central corneal thickness.

In nine of the studies, the mean and SD of central corneal thickness after PKP and DALK was reported. A total of 271 eyes were treated with PKP and 316 eyes with DALK. The mean age of the cases treated with PKP and DALK was 31.56 and 30.72, respectively. Furthermore, the follow-up duration was 24.87 and 20.81 months for PKP and DALK, respectively. Heterogeneity between the studies was significant (I2 = 85.9%, *p*-value < 0.001). According to the meta-analysis results with the help of the random-effects model, integrated mean differences (mean PKP – mean DALK) for the central corneal thickness were measured as –0.10 (pooled MD = –0.10, 95% CI: –0.57 – 0.37, *p*-value = 0.671). Figure 2 shows the forest plot of the meta-analysis.

#### Spherical equivalent (SE)

**Figure 3:**
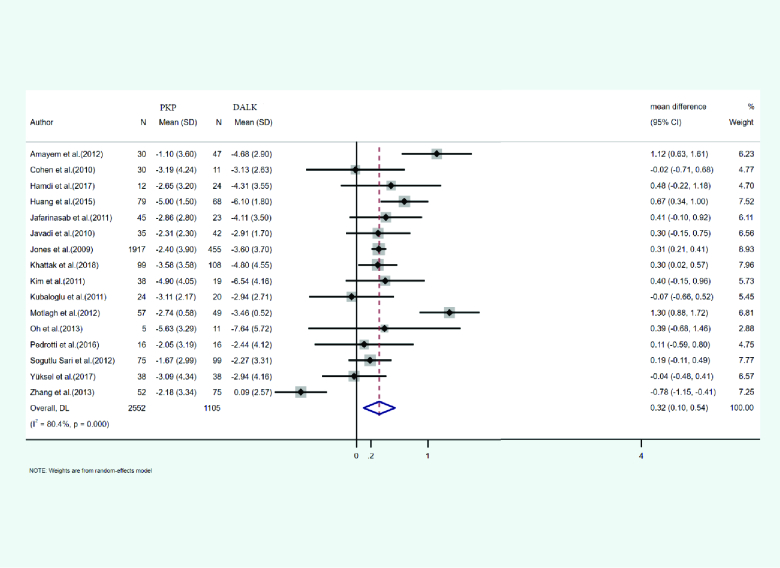
Results of the meta-analysis of the spherical equivalent.

In 16 studies, the mean and SD of the spherical equivalent identified after PKP and DALK was reported. A total of 2552 eyes were treated with PKP, and 1105 eyes were treated with DALK. The mean age of cases treated with PKP and DALK was 29.2 and 28.39 years, respectively. The duration of the follow-up was 35.36 months for PKP and 27.33 months for DALK cases. Heterogeneity was statistically significant (I2 = 80.4%, *p*-value < 0.001). Integrated mean differences (mean PKP – mean DALK) of PKP and DALK for the spherical equivalent was 0.32 (pooled MD = 0.32, 95% CI; 0.10 – 0.54, *p*-value = 0.004). Figure 3 illustrates the forest plot of the meta-analysis results.

#### Best-corrected visual acuity (BCVA)

**Figure 4:**
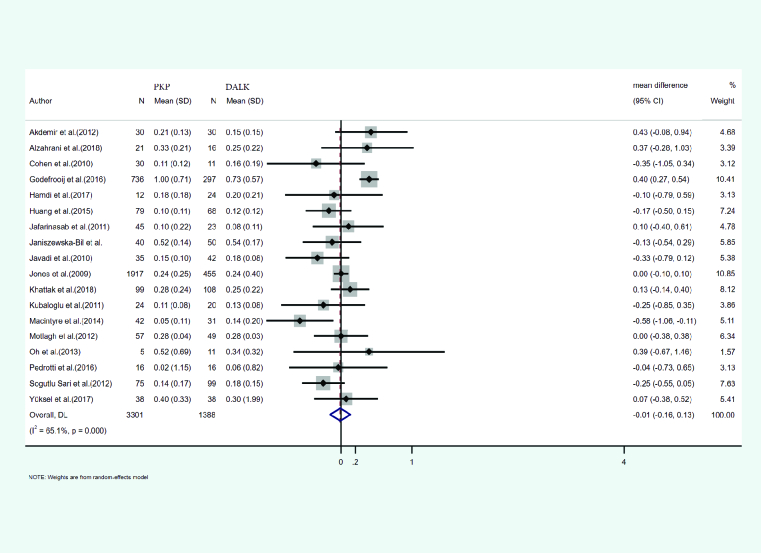
Results of the meta-analysis of the best-corrected visual acuity.

Eighteen studies reported the mean and SD of BCVA after both PKP and DALK. A total of 3301 eyes were treated with PKP and 1388 eyes with DALK. The mean age of cases treated with PKP and DALK was 30.54 and 28.14 years, respectively. Duration of follow-up was 29.71 months for PKP and 27.60 months for DALK cases. Heterogeneity was statistically significant (I2 = 65.1%, *p*-value < 0.001). Integrated mean differences (mean PKP – mean DALK) for the BCVA was measured as –0.01 (pooled MD = –0.01, 95% CI; –0. 61 – 0.13, *p*-value = 0.869). Figure 4 illustrates the forest plot of the meta-analysis.

#### Topographic cylinder

**Figure 5:**
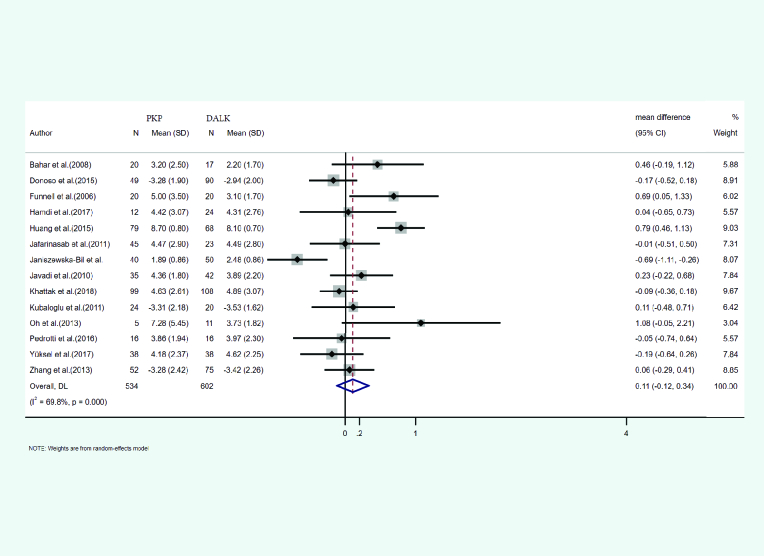
Results of the meta-analysis of the topographic cylinder.

The mean and SD of the topographic cylinder occurring after PKP and DALK was reported in 14 studies. A total of 534 eyes underwent PKP and 602 underwent DALK. The mean age of cases treated with PKP and DALK was 30.00 and 28.01 years, respectively. Follow-up for PKP and DALK groups was 35.61 and 28.79 months, respectively. Heterogeneity was statistically significant (I2 = 69.8%, *p*-value < 0.001). Integrated mean differences (mean PKP – mean DALK) for the topographic cylinder was 0.11 (pooled MD = 0.11.95% CI; –0.12 – 0.34, *p*-value = 0.359). Figure 5 shows the forest plot of the meta-analysis.

#### Refractive cylinder

**Figure 6:**
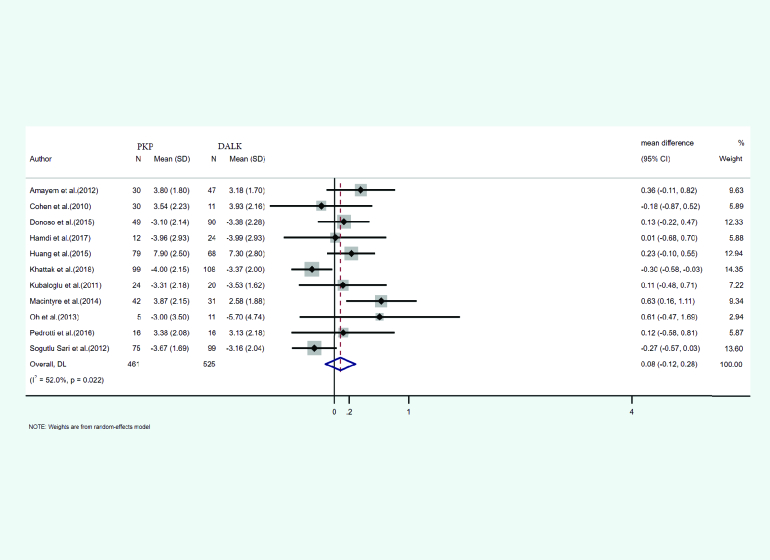
Results of the meta-analysis of the refractive cylinder.

The mean and SD of refractive cylinder occurring after PKP and DALK was reported in 11 studies. A total of 439 eyes underwent PKP and 525 underwent DALK. The mean age of cases treated with PKP and DALK was 29.65 and 29.09 years, respectively. Follow-up for PKP and DALK groups was 38.57 and 34.91 months, respectively. Heterogeneity was statistically significant (I2 = 52%, *p*-value = 0.022). Integrated mean differences (mean PKP – mean DALK) for the refractive cylinder was 0.08 (pooled MD = 0.27, 95% CI; –0.12 – 0.28, *p*-value = 0.428). Figure 6 shows the forest plot of the meta-analysis.

#### High IOP

High IOP occurring after PKP and DALK appeared in eight studies. A total of 349 eyes were treated with PKP, and 424 were treated with DALK. The mean age of cases treated with PKP and DALK was

**Figure 7:**
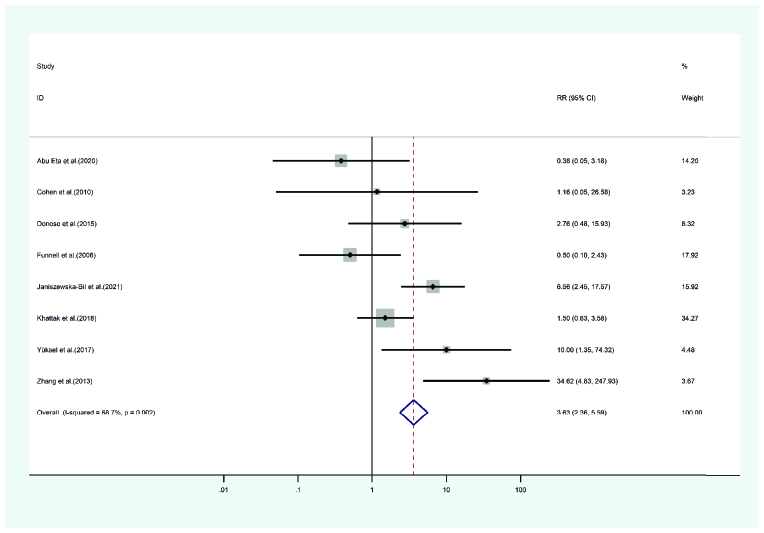
Results of the meta-analysis of the high IOP.

**Figure 8:**
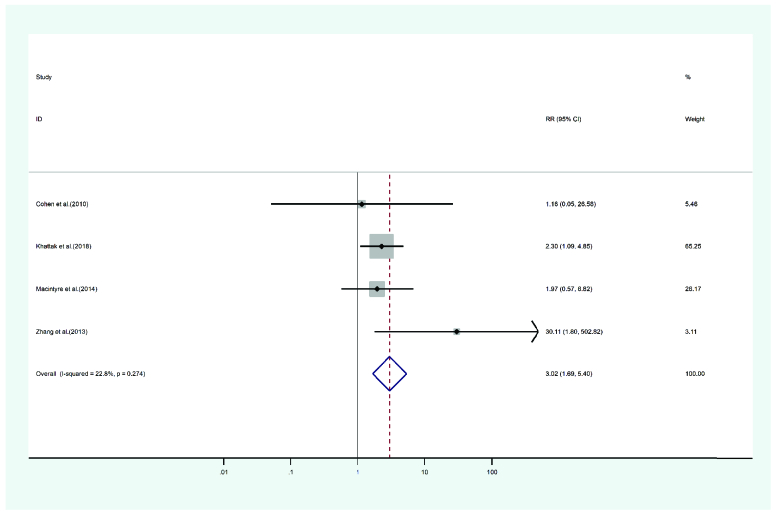
Results of the meta-analysis of the cataract.

**Figure 9:**
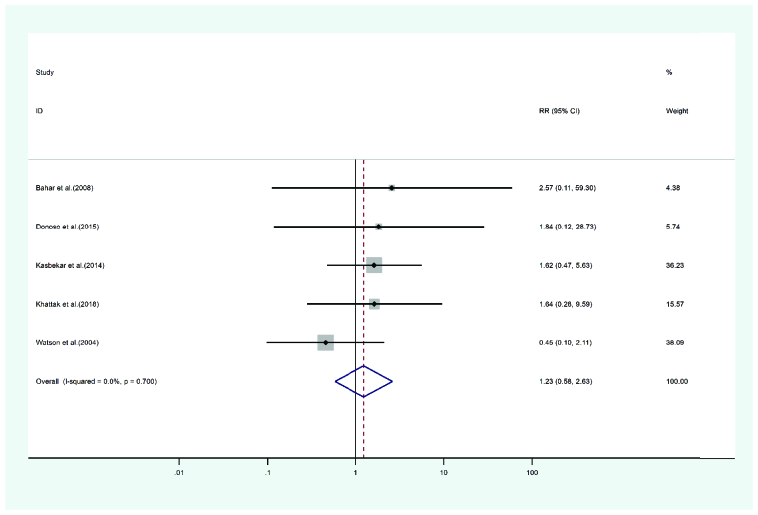
Results of the meta-analysis of the corneal infection.

**Figure 10:**
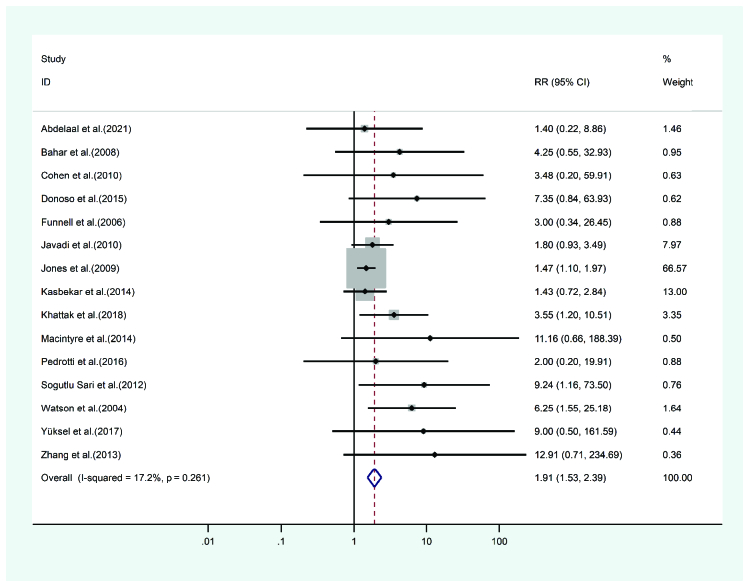
Results of the meta-analysis of the graft rejection.

**Figure 11:**
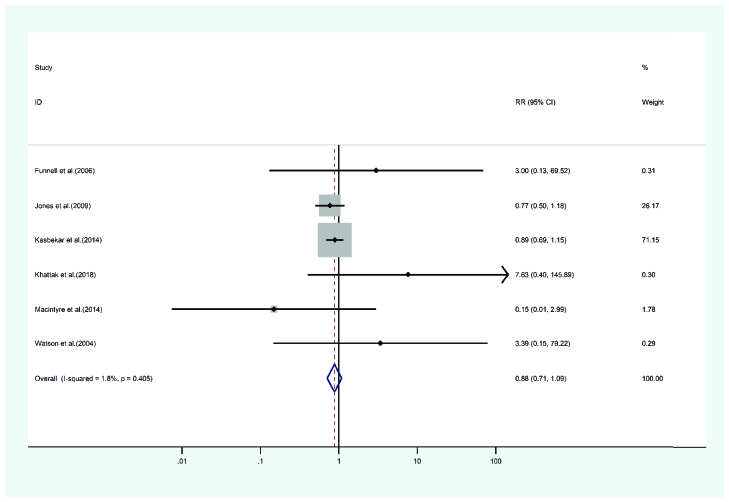
Graft failure meta-analysis results.

**Figure 12:**
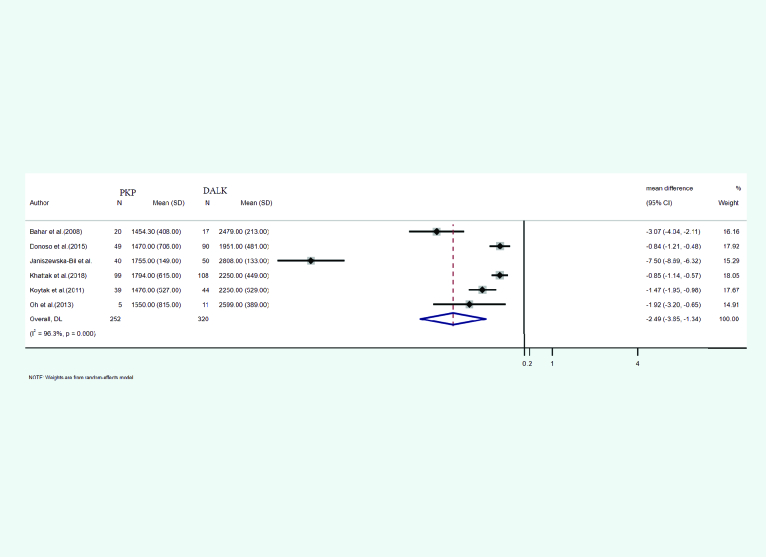
Endothelial cell count meta-analysis results.

30.51 and 30.52 years, respectively. The duration of follow-up for the PKP cases was 28 and 24.32 months for the DALK cases. Heterogeneity was statistically significant (I2 = 68.7, p-value = 0.002). The random-effects model showed the PKP group's high IOP risk ratio as 3.63 times that of the DALK group (pooled HR = 3.63, 95% CI; 2.36 – 5.59, *p*-value = 0.018). Forest's plot is presented in Figure 7.

#### Cataract

In six studies reporting cataract occurring after PKP and DALK, 223 eyes were treated with PKP and 225 eyes with DALK. The mean age of cases was 29.62 years for PKP and 30.77 years for DALK. Follow-up period was 41.27 months for PKP and 37.32 months for DALK cases. Heterogeneity was not statistically significant (I2 = 22.8, *p*-value = 0.274). The risk ratio of cataract incidence in the PKP group was 3.02 times that of the DALK group (pooled HR = 3.02, 95% CI; 1.69 – 5.40, *p*-value = 0.62). Figure 8 presents the forest plot of the meta-analysis.

#### Corneal infection

Corneal infection manifesting after PKP and DALK was reported in five studies. A total of 3314 eyes were treated with PKP and 1326 eyes were treated with DALK. The mean age of cases treated with the PKP and DALK method was 34.17 and 30.3 years, respectively. The duration of the cases' follow-up in the PKP and the DALK groups was 49.22 and 33.98 months. Heterogeneity was not statistically significant (Q-value = 0.24, df = 2, (I2 = 0.000, *p*-value = 0.7). The risk ratio of corneal infection incidence in the PKP group was measured at 1.23 times that of the DALK group (pooled HR = 1.23, 95% CI; 0.58 – 2.63, *p*-value = 0.700). Figure 9 illustrates the forest plot of the meta-analysis.

#### Graft rejection

Graft rejection occurring after PKP and DALK was observed in 15 studies. A total of 5554 eyes were treated with PKP and 2134 eyes with DALK. The mean age of cases undergoing PKP was 32.25 and 30.81 years for DALK. PKP cases were followed-up for 38 months and the DALK cases for 29.88 months. Heterogeneity was not statistically significant (I2 = 17.2, *p*-value = 0.261). The graft rejection risk ratio of PKP to DALK was 1.91 (pooled HR = 2.33, 95% CI; 1.69 – 3.22, *p*-value = 0.179). Figure 10 presents the forest plot.

#### Graft failure

Six studies discussed graft failure presenting after PKP and DALK. A total of 5224 eyes were treated with PKP and 1725 were treated with DALK. The mean age of cases treated with PKP and DALK was 31.77 and 29.4 years, respectively. Follow-up for the PKP group was 42 months and 35.98 months for the DALK group. Heterogeneity was not statistically significant (I2 = 1.8, *p*-value = 0.405). The risk ratio of the graft failure incidence in the PKP group was 0.88 of the DALK group (pooled HR = 0.88, 95% CI; 0.71 – 1.09, *p*-value = 0.98). In Figure 11, a related forest plot is illustrated.

#### Endothelial cell count

The mean and SD of endothelial cell count presenting after PKP and DALK was reported in six studies. A total of 252 eyes underwent PKP and 320 underwent DALK. The mean age of cases treated with PKP and DALK was 32.47 and 29.95 years, respectively. Follow-up for PKP and DALK groups was 35.35 and 24.65 months, respectively. Heterogeneity was statistically significant (I2 = 96.3%, *p*-value < 0.001). Integrated mean differences (mean PKP – mean DALK) for the endothelial cell count was –2.49 (pooled MD = –2.49 95% CI; –3.65 – –1.34, *p*-value = 0.000). Figure 12 shows the forest plot of the meta-analysis.

##  DISCUSSION

In this study, a total of 6773 cases of PKP and 2891 cases of DALK were documented and reviewed. In those studies, where differences in sex were evaluated, the male cases were subjected to more interventions in both the PKP and DALK procedures than the female cases. The mean age for PKP and DALK categories was 30.97 and 29.80 years, respectively. Furthermore, the cases were followed-up in the PKP and DALK groups for 34.99 and 28.59 months, respectively.

CCT in the DALK group was 10% greater overall than registered in the PKP group, which was not statistically significant. The study by Liu et al^[53]^ could not find statistical significance regarding the differences in this value when comparing the two groups.

The SE value in the PKP group was 32% higher overall, which was also statistically significant. This finding may be due to the tighter suturing in DALK. Henein et al ${}^{[}$ ${}^{54]}$ and Liu et al^[53]^ did not find any statistical significance when comparing the value between the two groups. However, the study by Song et al^[55]^ found this value to be significantly more improved in the DALK group.

BCVA measured in the DALK group insignificantly demonstrated to be 1% better as compared to the PKP group. However, the study by Henein et al^[54]^ revealed statistical significance in favor of the PKP group. In Song et al^[55]^ and Liu et al^[53]^, this value was not statistically significant.

Regarding the topographic cylinder, the PKP group showed insignificantly greater results (about 11%). Furthermore, neither of the studies by Henein et al^[54]^ nor Song et al^[55]^ demonstrated statistical significance.

The PKP group demonstrated insignificantly greater results on the refractive cylinder values than the DALK group (8% overall). Henein et al^[54]^ demonstrated significance in the improvement of the RC in the DALK group. Song et al,^[55]^ however, found no statistical significance in the differences of RC between the two groups.

The ECC was 2.49 score unique higher in the DALK group as compared to the PKP group. Consistent with the study by Liu et al,^[53]^ this finding also turned out to be statistically significant. In the study by Henein et al,^[54]^ the ECC values were not statistically significant between the two groups. DALK involved the inner portion of the cornea less often than PKP. The procedure is also less invasive.

Cataracts (38 vs 12 cases), graft rejection (413 vs 80 cases), graft failure (286 vs 105 cases), High-IOP (73 vs 30 cases), and corneal infection (22 vs 11 cases) were all more common findings in the PKP groups as compared to the DALK groups. Except for the high IOP, cataract and graft rejection the remaining complications were not statistically significant when we compared the results of the two groups.

Graft failure, consistent with our study, was not statistically significant in the studies by Henein et al^[54]^ and Liu et al.^[53]^ In addition, High IOP was significantly more common in the PKP group when compared in the study by Liu et al.^[53]^ Furthermore, in prior review studies by Henein et al,^[54]^ Song et al,^[55]^ and Liu et al,^[53]^ the DALK groups suffered significantly fewer graft rejection episodes. Furthermore, consistent with our study, Liu et al^[53]^ found statistical significance regarding the number of post-op cataracts occurring in the PKP group.

##  SUMMARY

Despite the results favoring the DALK procedure and its utility in most of the evaluated outcomes, we cannot definitively conclude that the procedure is more eventful compared to PKP. This remark is primarily due to the small sample size, study design variability, and mismatched follow-up durations leading to, in some incidences, significant heterogeneity that could not be addressed via met-regression or sensitivity analyses. Therefore, we believe that any conclusions from the comparisons must be taken with a grain of salt. Ultimately, we believe that to increase the validity of a possible meta-analysis, future randomized controlled trials need to be conducted with consistently matching follow-up durations and timing between the sessions.

##  Limitations

Our study's limitation was that we did not include other types of lamellar keratoplasty techniques due to the low number of available cases. Furthermore, formulas used to convert the domains to the SD might not be as accurate as desired, which can be due to statistical limitations.

##  Financial Support and Sponsorship

Nil.

##  Conflicts of Interest

The authors do not have any conflicts of interest.
